# Reliability, validation and norms of the Chinese version of Anxiety Sensitivity Index 3 in a sample of military personnel

**DOI:** 10.1371/journal.pone.0201778

**Published:** 2018-08-09

**Authors:** Wenpeng Cai, Wei Dong, Yu Pan, Cun Wei, Shuimiao Zhang, Bin Tian, Jin Yan, Guanghui Deng

**Affiliations:** 1 Department of Psychology and Mental Health, Second Military Medical University, Shanghai, China; 2 Department of Medical Psychology, General Hospital of PLA, Beijing, China; 3 Department of Neurology, Jinan Military General Hospital of PLA, Jinan, China; University of Wuerzburg, GERMANY

## Abstract

This study aimed to explore the properties of the Chinese version of the Anxiety Sensitivity Index– 3 (ASI-3) in a sample of military personnel. Using non-probabilistic sampling, the Chinese version of the ASI-3 was administered to 3,077 valid participants aged 16 to 36 years old (M = 22.35, SD = 3.57) from nine military units. The Depression Anxiety Stress Scales-21 (DASS-21) and The State-Trait Anxiety Inventory (STAI) were used to assess the construct validity. A one-way analysis of variance was conducted to compare the differences in the services and positions of the participants. It was found that ①The indices of confirmatory factor analysis met the standard values, which supported the hypothesis of the three-factor model of the original ASI-3; ②ASI-3 was significantly associated with DASS-21 in positive ways, which indicated the high convergent validity; on the other hand, the correlation between ASI-3 and TAI was relatively low, which indicated there was an empirical discrimination between anxiety sensitivity and trait anxiety. ③The Cronbach’s α coefficients were 0.926 for the total scale and 0.828–0.841 for the three subscales; ④At a cut-off score of 16, the sensitivity and specificity levels were 71.1% and 76.7%, respectively, where the sum of sensitivity and specificity becomes the maximum, accompanied with improvement of PPV and NPV; ⑤There were significant findings in the ASI and subscales among the five services and four positions. This study provides new evidence that the Chinese version of the Anxiety Sensitivity Index-3 has good validity and reliability and could be applied as an effective tool to assess anxiety sensitivity in military personnel. Our recommendations to researchers and practitioners are that the three factor model should be replicated across some different special forces and the items and constructs could be modified on Chinese culture.

## Introduction

Within the cognitive-behavioral framework, dysfunctional beliefs play an important role in emotional disorders [1]. For example, social phobia is considered to be affected by exaggerated beliefs that are viewed as threatening (e.g., when I tremble in the presence of others, I fear what people might think of me)[2]. Panic disorder is considered to be influenced by catastrophic beliefs of the danger of internal sensations (e.g., When I notice my heart skipping a beat, I worry that there is something seriously wrong with me) [3]. These dysfunctional cognitions cause catastrophic misinterpretations of danger cues, thereby triggering situational anxiety and fear [4]. Specifically, anxiety sensitivity (AS) is thought to be a cognitive diathesis including a set of trait-like dysfunctional beliefs about harmful consequences of anxious arousal.

Reiss and McNally defined anxiety sensitivity as “the fear of arousal-related sensation, arising from beliefs that the sensations have harmful somatic, cognitive, or social consequences (e.g., death, insanity, or social rejection)” [5]. As individuals with high anxiety sensitivity feel anxiety, they must be alarmed by the arousal-related sensations, which give rise to an intensification of anxiety symptoms, fear response, and avoidance behaviors. A large body of research suggests that AS is a strong psychological risk factor for the development and maintenance of anxiety psychopathology [6, 7]. Additionally, existing studies indicate that anxiety sensitivity might be associated with several psychosomatic diseases, such as hypochondriasis, chronic pain, and substance use disorders [8].

There is a similar construct Trait Anxiety, which also reflects individuals’ sensitivity to develop anxious mood. However, trait anxiety is deemed to be the tendency to respond fearfully to various stressors, and anxiety sensitivity refers to a proneness to respond with anxiety to anxiety symptoms[9]. Although fear and anxiety often occur together, this does not mean that these terms are interchangeable as theoretical terms. Anxiety is a rather vague feeling of discomfort while fear is triggered by imminent danger, so that anxiety is driven by uncertainty about anticipated events while fear is the response to certain imminent events. Reiss noted that while trait anxiety predicts future anxiety based on anxiety experiences in the past, AS predicts future anxiety based on the beliefs assessed by the ASI, regardless of the frequency of anxiety experiences in the past [10]. Trait anxiety cannot explain why some people react with anxiety to their own anxiety or anxiety related sensations[11]. One the other hand, Reiss and McNally found that correlations between the ASI and trait anxiety have been consistently lower than the correlations between trait and state anxiety[12, 13]. Taylor and Cox pointed out a modest correlation (r = 0.26) between trait version of STAI and 60 item-Anxiety Sensitivity Profile which is a revised form of the ASI, and concluded that AS and trait anxiety are correlated but distinct factors[14, 15].

The original Anxiety Sensitivity Index (ASI) was composed of 16 items that assess various components of the fear of anxiety-related sensations [16]. A high internal consistency (Cronbach’s α = 0.82–0.88) and acceptable test-retest reliability (14 days, r = 0.75) were verified in two studies [17, 18]. Several factor analytic studies showed 60% of the total variance could be explained by three factors, including fears of negative consequences of arousal-related physical, cognitive, or social sensations [19, 20].

Therefore, Taylor and Cox developed the 36-item Anxiety Sensitivity Index–Revised (ASI-R), which was supposed to assess the following six lower-order factors in greater depth: (a) cardiovascular fears, (b) respiratory fears, (c) gastrointestinal symptoms and fears, (d) fear of cognitive dyscontrol, (e) fear of publicly observable anxiety reactions, and (f) neurological and dissociative symptoms.[8] However, the ASI-R failed to obtain stable factor structures, given the different solutions concluded by different studies [21, 22]. A psychometrically sound, multidimensional measure of AS was supposed to be developed in the following studies.

In response to these concerns, the 18-item Anxiety Sensitivity Index-3 was developed by Taylor et al. to improve the basic psychometric criteria of the original scales [23]. Three subscales of physical, cognitive, and social concerns were included in the ASI-3, which showed strong correlations with (r ≥ 0.5) and higher internal consistency the (Cronbach’s α ≥ 0.73) the 16-item ASI and explained approximately 76% of the total variance.

With the advantage of the structure and a potential relationship with anxiety disorders, the ASI-3 has attracted many researchers’ attention. To date, several cross-cultural studies have contributed to a more universal etiological model of anxiety sensitivity and related anxiety disorders [24–27]. Likewise, many Chinese researchers are devoted to demonstrating the role of AS in the development and maintenance of mental disorders [28–30]. Li et al. translated the ASI-R and assessed its reliability and validity in a sample of middle school students [31]. Then, Wang et al. revised the Chinese version of the ASI-3 and formulated the ASI-3 norm of Chinese healthy adult women [32]. However, many specific psychometric characteristics remained to be assessed in a larger sample, especially in males, because several studies showed that gender differences existed in anxiety sensitivity and its subscales [20, 33]. Although the majority of soldiers are not engaged in war or conflicts, they have to face many other types of stressors, such as training exercises, heavy workloads, and family separations [34, 35]. These inevitable stressors could give rise to anxiety, depression, and posttraumatic stress disorder [36], as well as suicidal behaviors [37, 38], which might deeply impair their functions in social, occupational and other significant fields [39]. Therefore, military psychologists were supposed to pay attention to how to distinguish anxiety-sensitive individuals and reduce the effect of anxiety disorders on military task effectiveness.

Above all, the current study was aimed to investigate the reliability, validity and norms of the ASI-3 in a sample of Chinese military personnel and address the following hypotheses (a) whether the three factor model could be replicated in current samples (factorial validity); (b) the relationship between AS and other anxiety constructs (construct validity); (c) the split-half model and Cronbach's alpha coefficient of ASI-3 (within-session reliability); (d) the cut-off score to predict anxious individuals (ROC curve); (e) the differences of the ASI-3 and its subscales in terms of services and positions.

## Materials and methods

### Participants

The participants were selected from nine military units using non-probabilistic (convenience) sampling, covering all five major components (Army, Navy, Air Force, Armed Police and Rocket Force) and five theater commands (Eastern, Southern, Western, Northern and Central)[40]. Finally, a total of 3,077 valid participants were included and were aged 16 to 36 years (*M* = 22.35, *SD* = 3.57); 94.0% of the participants were male. Additional demographic variables can be found in Table 1. The participants were directed to attend a large questionnaire-based study, which lasted approximately 15 min. They were instructed to read and sign the informed consent on the first page of the questionnaires, then were asked to complete the paper-and-pencil questionnaires in a small group.

**Table 1:** Demographic variables by all the participants.

Item		n(%)
Gender	Male	2893(94.0)
	Female	184(6.0)
Only child in family	Yes	1176(38.2)
	No	1901(61.8)
Marital status	Unmarried	2255(73.3)
	Married	788(25.6)
	Divorced	34(1.1)
Education level	Low (≤12 years)	2163(70.3)
	High (>12 years)	914(29.7)
Position	Soldier	1410(45.8)
	Sergeant	746(24.2)
	Officer	572(18.6)
	Cadet	349(11.3)
Service	Amry	1079(35.1)
	Navy	800(26.0)
	Air Force	464(15.1)
	Armed Police	361(11.7)
	Rocket Force	373(12.1)

### Measures

*The Anxiety Sensitivity Index-3 (ASI-3)* [23] includes 18 items that assess anxiety-related physical (e.g., “When my stomach is upset, I worry that I might be seriously ill”), cognitive (e.g., “When my thoughts seem to speed up, I worry that I might be going crazy”), or social (e.g., “It scares me when I blush in front of people”) concerns. Participants were asked to rate how much they share these concerns on a 5-point scale (0 = *I agree very little*, 4 = *I agree very much*). The Chinese version showed a good reliability (Cronbach’s α = 0.95) in healthy Chinese women and correlated moderately with Beck Anxiety Inventory (Pearson’s *r* = 0.32 to 0.42)[32].

*The Depression*, *Anxiety*, *and Stress Scales-21 (DASS-21)*[41] is a self-report tool concluding 21 items to assess three constructs: Depression, Anxiety and Stress. Participants were asked to rate how much they share these concerns (e.g., “I was worried the about situations in which I might panic”) on a 4-point scale (0 = *Did not apply to me at all*, 3 = *Applied to me very much or most of the time*). The Chinese version showed high validity and reliability in Chinese college students[42].

*The State-Trait Anxiety Inventory (STAI)* [43] is one of the most widely used scales to assess individuals’ state and trait anxiety (e.g., ‘‘I worry too much over something that really doesn’t matter”). The items used a 4-point Likert scale ranging from 1 (almost never) to 4 (almost always). The Chinese version also showed high validity and reliability [44]. Only the trait anxiety inventory (TAI) was used in the current study and 2277 participants finished it.

### Statistical analysis

The percentage of missing data was 3.6% and assumed to follow an arbitrary missing pattern. Therefore, Markov Chain Monte Carlo multiple imputation was used to replace missing data, which was reported to be a preferred technique for handling missing values due to several advantages over other approaches[45]. The main analysis included the factorial validity of the 18-item ASI-3, which was assessed through structural equations (SEM) using confirmatory factor analysis (CFA). The model was estimated using AMOS17.0. Goodness-of-fit was assessed with several robust fit indexes [46, 47], including the ratio of chi-square to the degree of freedom (χ^2^/df), the comparative fit index (CFI), the Tucker-Lewis index (TLI), and the root-mean-square error of approximation (RMSEA). The acceptability criteria for these indicators were as follows: χ^2^/df less than 5 [48], both CFI and TLI greater than 0.90 [47], and RMSEA less than 0.08 [49].

An estimated internal consistency reliability (Cronbach’s alpha and item-factor correlation) was assessed in the current study, which meant the degree as to which a score or sub-score of a questionnaire reflected a narrow/ closely-confined or rather broad/widely-ranged construct[50, 51]. Besides, a stability test was conducted based on the Guttman split-half coefficient, which was also important to determine psychometric quality and test reliability[52]. What’s more, Multi-Trait-Multi-Method (MTMM) was conducted to assess the construct validity of the ASI-3 against the DASS-21 and TAI. According to Campbell and Fiske[53], MTMM could provide a practical methodology that researchers and practitioners could actually use for purposes of construct validation, especially convergent and discriminant validation. Previously, correlations from 0.40 to 0.70 have been considered satisfactory for convergent validity[54]. If correlation is too high (>0.70), it can be questioned whether the scales really assess different concepts and whether the application of two highly correlated scales adds useful information compared to if only one of the scales in included. Conversely, by discriminate validity one assumes that scales assessing different constructs should show low correlation (<0.40)[54, 55]. Additionally, a receiver operating characteristic (ROC) analysis was conducted to ascertain the cut-off scores for correctly identifying soldiers with anxiety (defined as a DASS-21 Anxiety score >7) [42]. The Delong Clarke-Pearson method was used to compute the areas under the ROC curves (AUCs) [56]. Lastly, one-way analysis of variance followed by LSD post-hoc test for multiple groups comparison was used to compare the AS differences in the services and positions of the participants. SPSS for Windows version 23.0 (SPSS Inc., Chicago, IL, USA) was used to conduct the above statistical analyses.

### Ethics statement

The present study protocol was reviewed and approved by the institutional review board of the Second Military Medical University (IRB No. 20152049). All the participants were adults and signed the informed consent approving the use of their data for research purposes. For participants from 16 to 18 years old, the ethics committee has approved the lack of parent or guardian consent.

## Results

### Descriptive analysis

Population parameters (M, SD, skewness, and kurtosis) of ASI are displayed in Table 2. According to Kline, the cut-off of the absolute values of skewness and kurtosis were 3.0 and 8.0, respectively [57]. In the current study, the univariate skewness for scores on the measurement instruments ranged from 0.784 to 2.175, and the univariate kurtosis for the instruments ranged from 0.037 to 5.756. Both met the acceptability criteria of normal distribution, so no adjustments were made to the scores on the variables in the following analysis.

**Table 2:** Population parameters of ASI-3.

Item		ASI-3				Physical Concerns				Social Concerns				Cognitive Concerns			
		*M*	*SD*	Skewness	Kurtosis	*M*	*SD*	Skewness	Kurtosis	*M*	*SD*	Skewness	Kurtosis	*M*	*SD*	Skewness	Kurtosis
Gender	Male	9.64	10.464	1.597	2.907	2.36	3.370	2.022	4.829	4.76	4.608	1.157	1.020	2.51	3.541	1.944	4.194
	Female	7.63	8.171	1.686	2.931	1.84	2.681	1.967	3.841	3.66	3.482	1.062	0.580	2.13	2.972	2.175	5.428
Only child in family	Yes	10.19	10.560	1.492	2.354	2.48	3.476	1.912	4.128	4.96	4.560	1.081	0.771	2.75	3.654	1.784	3.318
	No	9.10	10.201	1.697	3.455	2.24	3.242	2.112	5.449	4.54	4.546	1.233	1.307	2.33	3.409	2.085	5.038
Marital status	Unmarried	9.62	10.461	1.632	3.176	2.35	3.353	2.028	4.968	4.77	4.584	1.149	1.042	2.50	3.548	1.999	4.513
	Married	9.29	10.144	1.541	2.250	2.31	3.330	2.017	4.484	4.52	4.494	1.249	1.231	2.47	3.446	1.819	3.363
	Divorced	7.65	7.356	1.132	0.921	1.41	1.861	1.542	1.908	4.24	4.046	0.787	0.037	2.00	2.202	1.339	1.593
Education level	Low (≤12 years)	8.47	10.197	1.821	3.856	2.14	3.264	2.175	5.756	4.05	4.354	1.426	2.035	2.28	3.502	2.114	4.956
	High (>12 years)	12.01	10.294	1.310	1.938	2.79	3.456	1.773	3.466	6.24	4.654	0.784	0.096	2.98	3.481	1.677	3.163

### Validity

Our study aimed to establish the factorial validity of the Chinese version of the Anxiety Sensitivity Index 3 in a sample of military personnel. Therefore, a confirmatory factor analysis was performed to examine the three-factor model. Overall, the fit indices mainly supported the scale structures: χ^2^/df = 16.583 (*P*<0.001), CFI = 0.925, TLI = 0.901, RMSEA = 0.071 and managed to meet the standard values.

**Fig 1:**
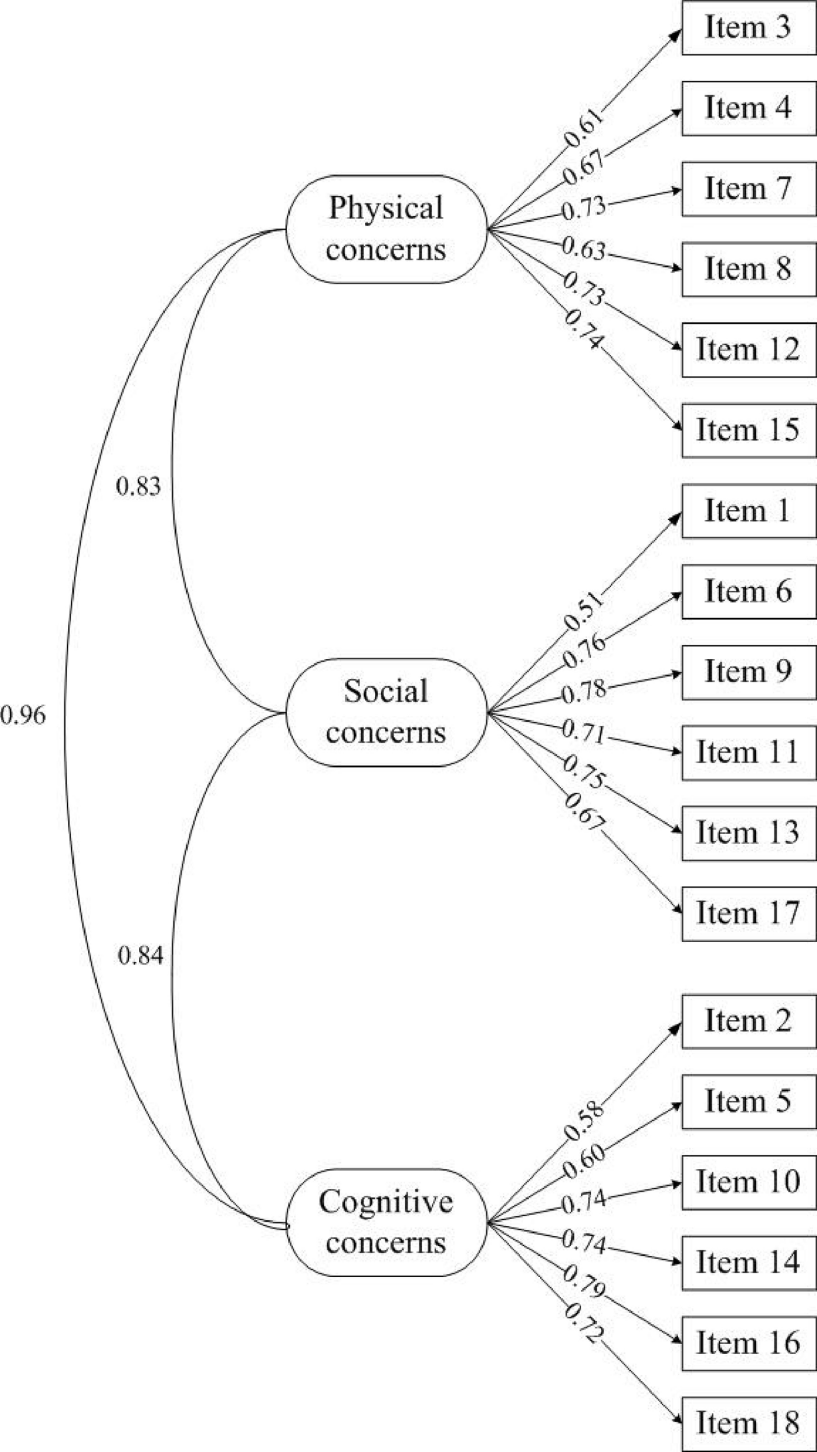
Factor structure of the ASI-3—The values between item and subscale is factor loading; The values between different scales is correlation coefficient.

A further detailed examination of the factor loadings showed the information on the analytical fit of the model, complementing the overall fit information. All indicators significantly loaded (*P*<0.01) on the hypothesized factor, supporting the adequacy of the three-factor model. As Fig 1 shows, the standardized factor loadings ranged from a minimum of 0.51 (item 1: It is important to me not to appear nervous) to a maximum of 0.79 (item 16: When I have trouble thinking clearly, I worry that there is something wrong with me). Most of the factor loading was better than those in a previous study using a sample of healthy adult women [32].

Additionally, the MTMM correlation matrix is shown in Table 3. The total score and subscale scores correlated with each other (r = 0.700–0.913, p<0.001) and were also significantly associated with DASS-21 in positive ways (r = 0.432–0.597, p<0.001). This finding indicated that the convergent validity of the ASI-3 with related variables met the standard values in the current sample. One the other hand, correlation between ASI-3 and TAI was relatively low (r = 0.169–0.290, p<0.001), which indicated there was an empirical discrimination between anxiety sensitivity and trait anxiety.

**Table 3:** Multi-Trait-Multi-Method correlation matrix among ASI-3, DASS-21 and TAI.

	1	2	3	4	5	6	7	8
1.ASI-3	(0.926)							
2.Physical Concerns	0.900***	(0.828)						
3.Social Concerns	0.910***	0.700***	(0.835)					
4.Cognitive Concerns	0.913***	0.797***	0.720***	(0.841)				
5.DASS-21 Depression	0.524***	0.445***	0.432***	0.526***	(0.828)			
6. DASS-21Anxiety	0.597***	0.507***	0.522***	0.561***	0.812***	(0.818)		
7. DASS-21 Stress	0.577***	0.449***	0.544***	0.532***	0.793***	0.803***	(0.834)	
8.TAI	0.259***	0.260***	0.169***	0.290***	0.322***	0.386***	0.384***	(0.848)

Note. Cronbach’s alpha coefficients shown in the diagonal brackets.

***: p<0.001

### Reliability

Cronbach’s alpha coefficients were computed and are shown in the brackets of Table 2. Alpha coefficients had a value of 0.828 for the Physical concerns dimension (95% CI 0.818–0.837), 0.835 for the Social concerns dimension (95% CI 0.826–0.844), and 0.841 for the Cognitive concerns dimension (95% CI 0.833–0.850). Moreover, the internal consistency of the entire scale was 0.926 (95% CI 0.922–0.930). The Guttman split-half coefficient of ASI-3 was 0.881, indicating that it has adequate reliability.

Descriptive statistics, item homogeneity (item-factor correlation), and alpha if item deleted are presented in Table 4. Overall, the estimated internal consistency of the scale can be considered adequate.

**Table 4:** Means, standard deviations, item-factor correlation, alpha if item deleted for the items of the ASI-3.

	Mean	SD	Item-factor correlation	Alpha if item deleted
ASI-3	9.50	10.36		
Physical Concerns	2.33	3.34		
item 3	0.64	0.91	0.703	0.922
item 4	0.45	0.82	0.757	0.922
item 7	0.41	0.78	0.788	0.922
item 8	0.27	0.70	0.716	0.924
item 12	0.28	0.67	0.743	0.922
item 15	0.28	0.64	0.725	0.922
Social Concerns	4.70	4.56		
item 1	1.39	1.20	0.673	0.929
item 6	0.89	1.12	0.818	0.921
item 9	0.61	0.91	0.790	0.920
item 11	0.53	0.87	0.725	0.922
item 13	0.63	0.97	0.769	0.921
item 17	0.65	1.04	0.704	0.923
Cognitive Concerns	2.49	3.51		
item 2	0.46	0.81	0.715	0.923
item 5	0.60	0.86	0.718	0.923
item 10	0.40	0.83	0.763	0.921
item 14	0.24	0.62	0.733	0.922
item 16	0.43	0.80	0.818	0.920
item 18	0.35	0.78	0.752	0.922

### Cut-off scores for anxiety

**Fig 2:**
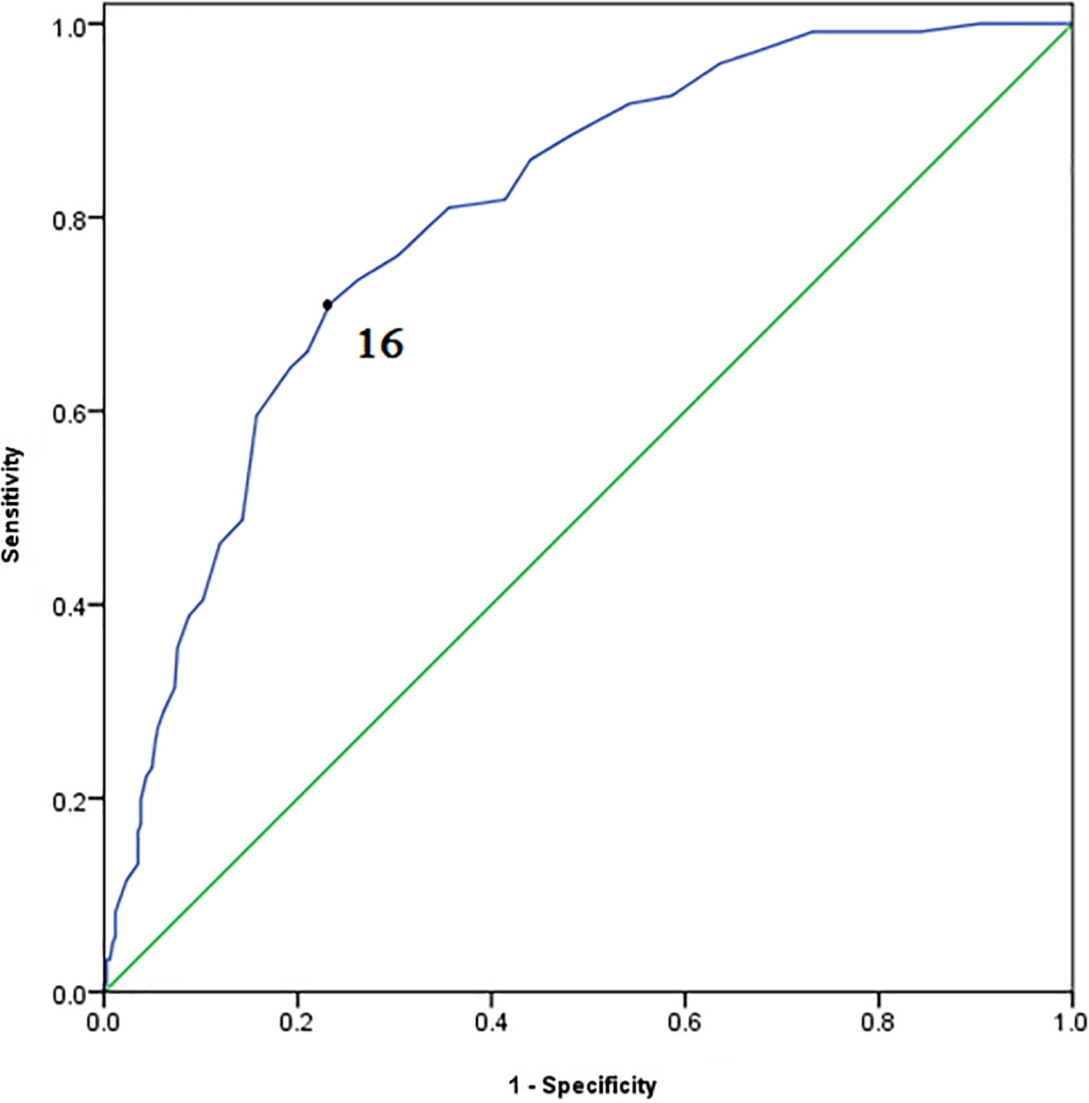
Receiver operating characteristics curve for the ASI-3 to detect anxious individuals.

Individuals with a score of 7 or higher on the DASS-21 Anxiety subscale were defined as anxious, based on the diagnostic performances of ASI-3, which are illustrated in Fig 2 and Table 5. Maximal discrimination between anxiety and non-anxiety was obtained at the cut-off score of 16, accompanied by the improvement of positive predictive value and negative predictive value. This result means that a score of 16 or higher indicates the presence of anxiety. When 16 was used as the cut-off value, the sensitivity, specificity, positive predictive value, negative predictive value, and Area Under the Curve were 71.1%, 76.7%, 51.8%, 88.3% and 0.800 (*P*<0.001; 95% CI: 0.757–0.843), respectively.

**Table 5:** Sensitivity, specificity, positive predictive value, and negative predictive value of ASI-3 at various cut-off scores for anxiety.

Score	Sensitivity, %	Specificity, %	Positive predictive value, %	Negative predictive value, %
≥14	76.0	69.7	46.9	89.2
≥15	73.6	73.8	49.7	88.8
****≥16****	****71.1****	****76.7****	****51.8****	****88.3****
≥17	66.1	79.0	52.6	86.9
≥18	64.5	80.8	54.2	86.6

### Differences according to services

As Table 6 shows, there were significant differences in the ASI and subscales among five services (F = 45.934–123.389, *P*<0.001). In the multiple comparisons, total points and subscales were different from each other (*P*<0.05), except for physical concerns between navy and air force (LSD-t = -0.345, *P* = 0.068), army and rocket force (LSD-t = -0.261, *P* = 0.180), social concerns between navy and rocket force (LSD-t = 0.123, *P* = 0.643), and cognitive concerns between navy and air force (LSD-t = -0.291, *P* = 0.144).

**Table 6:** Comparison of ASI and subscales among five services (Mean±SD).

	Army (n = 1074)	Navy (n = 800)	Air Force (n = 464)	Armed Police (n = 359)	Rocket Force (n = 373)	F (4, 3072)
Physical Concerns	1.77±2.686^▼■◆^	3.10±4.242^▲■◆●^	3.45±3.697^▲◆●^	1.14±2.119^▲▼■●^	2.03±2.410^▼■◆^	45.934***
Social Concerns	3.53±3.734^▼■◆●^	5.26±5.161^▲■◆^	7.96±4.681^▲▼◆●^	2.28±2.838^▲▼■●^	5.14±3.897^▲■◆^	123.389***
Cognitive Concerns	1.91±2.888^▼■◆●^	3.32±4.503^▲■◆●^	3.61±3.644^▲◆●^	1.04±1.861^▲▼■●^	2.37±2.874^▲▼■◆^	48.258***
ASI-3	7.17±8.378^▼■◆●^	11.68±12.918^▲■◆●^	15.01±10.657^▲▼◆●^	4.37±5.610^▲▼■●^	9.50±7.942^▲▼■◆^	85.307***

***: p<0.001.

▲indicates significant difference compared with Army

▼indicates significant difference compared with Navy

■indicates significant difference compared with Air Force

◆indicates significant difference compared with Armed Police

●indicates significant difference compared with Rocket Force. These significant differences are at the 0.05 level.

### Differences based on positions

As Table 7 shows, there were significant differences in the ASI and subscales among four positions (F = 19.544–102.758, *P*<0.001). In the multiple comparisons, no significant differences between soldier and cadet were observed in total points and subscales (*P*>0.05). Officers had higher scores in total points and subscales than individuals on the other three positions (*P*<0.001). Additionally, social concerns values were lower in sergeants than those in soldiers (LSD-t = -0.991, *P*<0.001) and cadets (LSD-t = -1.180, *P*<0.001). Soldiers scored higher on the ASI-3 than sergeants (LSD-t = 1.501, *P* = 0.001).

**Table 7:** Comparison of ASI and subscales among four positions (Mean±SD).

	Soldier (n = 1410)	Sergeant (n = 746)	Officer (n = 572)	Cadet (n = 348)	F (3, 3073)
Physical Concerns	2.20±3.396^■^	1.92±2.972^■^	3.31±3.729^▲▼◆^	2.33±2.810^■^	21.920***
Social Concerns	4.34±4.606^▼■^	3.35±3.738^▲■◆^	7.43±4.842^▲▼◆^	4.53±3.539^▼■^	102.758***
Cognitive Concerns	2.34±3.649^■^	2.09±3.276^■^	3.47±3.674^▲▼◆^	2.49±2.511^■^	19.544***
ASI-3	8.85±10.646^▼■^	7.35±9.224^▲■^	14.21±10.995^▲▼◆^	8.94±7.824^■^	54.989***

***: p<0.001.

▲ indicates significant difference compared with Soldier

▼indicates significant difference compared with Sergeant

■indicates significant difference compared with Office

◆indicates significant difference compared with Cadet. These significant differences are at the 0.05 level.

## Discussion

As the first Chinese large-sample norm, the current study aimed to provide evidence for the validity of the Chinese version of the ASI-3 using robust methods. More than three thousand soldiers in various services and positions were included, and the findings are summarized as follows.

First, the CFA results indicated that the Chinese version of the ASI-3 met basic psychometric quality criteria. Particularly, the three-factor (physical, social, and cognitive concern) model provided a good fit of the data structure, which indicated that a high construct validity was obtained in the current samples. In contrast, the fit indices tended to be poorer than those of several other cross-cultural studies [23]. This result may be due to the weakness of some items in the social and cognitive concerns subscale that showed lower loading in the Chinese version of the ASI-3 compared with the English version. However, the fit indices did not significantly improve, and these items were removed.

Besides, sufficient coefficient alpha values of ASI-3 and subscales (Table 2) indicated high internal consistency. Since test length has an effect on coefficient alpha values[50], r > 0.8 is quite high for the subscale with only 6 items. Meanwhile, the Guttman split-half coefficient of ASI-3 was 0.881, indicating that it has adequate reliability.

Additionally, slight correlations (r = 0.169–0.290) were seen between anxiety sensitivity and trait anxiety, which is another important neuroticism subcomponent to experiencing anxiety, which showed there is an empirical discrimination between anxiety sensitivity and trait anxiety. On the other hand, positive correlations between ASI and DASS-21, especially anxiety subscale, indicating high criterion-related validity of this instrument.

The AUC for identifying individuals with anxiety showed a high diagnostic accuracy on the ASI-3 (0.800). At a cut-off score of 16, the sensitivity and specificity levels were 71.1% and 76.7%, respectively, where the sum of sensitivity and specificity becomes the maximum, accompanied with improvement of PPV and NPV. Above results indicated that the ASI-3 had good criterion validity.

Interestingly, differences of AS in services and positions were found in the current study. Individuals in the Air Force tended to score higher than others on the ASI-3, especially on the social concerns subscale. According to a U.S. Air Force report, the suicide rate increased from 8.9 per 100,000 active duty Airmen in 2004 to 15.5 in 2012 [58]. Perceived lack of social support [59], low unit cohesion [60], and greater psychosocial difficulties influenced the severity of suicidal ideation among those Airmen affected [61]. Due to facing heavier workloads, more dangerous military tasks, and longer family separations, the Airmen suffered more from anxiety problems. Moreover, a strict elimination system in the AF led to the fear of showing anxiety in the presence of others, which also raised Airmen’s anxiety sensitivity and social concerns [62]. Among other military personnel, the officers reported much more anxiety sensitivity than others, and the sergeants reported the least, which was consistent with the findings reported by Jiang et al. of a sample of Chinese military personnel participating in an earthquake rescue [63]. Officers of ground soldiers’ shouldered many responsibilities, such as organizing trainings, ensuring safety, and executing commands, which required them to stay alert at all times. This responsibility directly gave rise to their anxiety sensitivity in the cognitive, physical, and social dimensions. In contrast, most sergeants were experienced soldiers who had adapted to military life. Their brain work was lighter than that of officers, and their manual work was lighter than that of soldiers. Therefore, they reported the lowest scores on the ASI-3 and its subscales.

There were still several limitations worthy of consideration. First, sample constructs remained to be improved. There were much important differences among sub-samples, thus future work should examine whether the three factor model could be replicated across difference samples, especially some special forces, such as airborne troops, submarine troops, and missile troops, who have to face many more stressors and might show poor mental health status. Second, the current study managed to investigate the reliability and validity of the 3-factor ASI in a sample of Chinese military personnel but failed to modify the items and constructs based on Chinese culture. Therefore, a more complex application of confirmatory factor analysis and cross-cultural methodology should be used in future research. Third, the cross-sectional design made it difficult to establish a causal relationship between the data. It remains to be determined whether high anxiety sensitivity individuals in the military would be more likely to develop an anxiety disorder in the battlefield environment or during retirement.

Nevertheless, the findings of the current study indicated that the Chinese version of the ASI-3 met the basic psychometric criteria. High construct validity, criterion validity, and internal consistency reliability suggested that the scale is useful in a sample of military personnel. Finally, the present findings provided strong support to focus on the emotional state of Airmen and officers and to take actions to improve their mental health.
